# Predictors of HIV prevalence among street-based female sex workers in Andhra Pradesh state of India: a district-level analysis

**DOI:** 10.1186/1472-6874-14-65

**Published:** 2014-05-06

**Authors:** G Anil Kumar, Rakhi Dandona, Michel Alary, Lalit Dandona

**Affiliations:** 1 Public Health Foundation of India, ISID Campus, 4 Institutional Area, Vasant Kunj, New Delhi 110 070, India; 2 URESP, Centre de recherche du CHU de Québec, Québec, Canada; 3 Département de médecine sociale et préventive, Université Laval, Québec, Canada; 4 Institute for Health Metrics and Evaluation, University of Washington, Seattle, WA, USA

**Keywords:** HIV, India, Street-based female sex worker, Contextual factors, Avahan

## Abstract

**Background:**

A decline in HIV prevalence among female sex workers (FSWs) has been reported from the Indian state of Andhra Pradesh between the two rounds of integrated biological and behavioural assessment (IBBA) surveys in 2005–06 and 2009, the first of these around the time of start of the *Avahan* HIV prevention intervention. In order to facilitate further planning of FSW interventions, we report the factors associated with HIV prevalence among street-based FSWs.

**Methods:**

Behavioural data from the two rounds of IBBA surveys, district-level FSW HIV prevention program data, and urbanisation data from the Census of India were utilized. A multilevel logistic model was used to investigate factors associated with inter-district variations in HIV positivity among street-based FSWs in the districts by fitting a two-level model.

**Results:**

The estimated HIV prevalence among street-based FSWs changed from 16% (95% confidence interval [CI] 14.2 – 17.7%) to 12.9% (95% CI 11.5 – 14.2%) from 2005–06 to 2009. HIV positivity was significantly higher in districts with a high proportion of FSWs registered with targeted interventions (odds ratio [OR] 2.02; 95% CI 1.18-3.45), and in districts with medium (OR 2.54; 95% CI 1.58-4.08) or high (OR 1.55; 95% CI 1.05-2.29) proportion of urban population. Districts which had met the condom requirement targets for FSWs had significantly lower HIV positivity (OR 0.50; 95% CI 0.26-0.97). In round 2 survey, the districts with medium level urbanisation had significantly higher proportion of FSWs registered with HIV intervention programmes and also reported higher consistent condom use with regular partner (p < 0.001).

**Conclusions:**

Variations in HIV positivity among street-based FSWs were seen at the district level in relation to HIV intervention programs and the degree of urbanization. These findings could be used to enhance program planning to further reduce HIV transmission in this population.

## Background

Female sex workers (FSWs) remain at a high risk for HIV infection in India, with sex work viewed as the primary means of transmission [1]. In particular, within FSWs the street-based FSWs are more vulnerable to HIV infection due to their social status and working conditions on the street [2-5]. Considerable attention has been given in India for prevention of HIV among FSWs over the last two decades through the National AIDS Control Organisation (NACO) and other programmes including *Avahan,* the India AIDS Initiative of the Bill and Melinda Gates Foundation [1,6,7], and a decline in HIV and STI prevalence and increase in condom use among FSWs has been reported [8-13].

The *Avahan* program was aimed at reducing HIV prevalence among high risk groups in India including FSWs in geographic areas most affected including those in the Indian state of Andhra Pradesh [6]. Two independent rounds of Integrated Behavioural and Biological Assessment (IBBA) surveys were conducted to measure the potential impact of *Avahan* interventions on HIV prevalence and related risk behavior among FSWs [14]. An overall declining trend in HIV prevalence among FSWs is reported from the state of Andhra Pradesh as part of the *Avahan* program [10]. An increase in consistent condom use with clients of FSWs between 2005–06 and 2009 has also been reported from the IBBA states [10,12,13]. A beneficial effect in reducing HIV prevalence at the population level over 5 years of *Avahan* program implementation in some of the states including Andhra Pradesh has also been reported [15,16].

Recent research has increasingly recognized the relevance of contextual factors that determine risk of HIV, and of structural interventions in improving outcomes of HIV interventions by changing the social, economic, political or environmental factors that determine risk and vulnerability [17-20]. In this background, we assessed certain contextual risk factors for HIV prevalence among street-based FSWs in Andhra Pradesh, along with individual risk factors. We also examined the effect of *Avahan* program indicators on HIV prevalence in the second round of IBBA. We studied street-based FSWs as they comprise the highest proportion among FSWs in Andhra Pradesh, and they are particularly exposed to HIV risk through their work on the street.

## Methods

Street-based FSWs were defined as FSWs aged 18 years or more who primarily solicited the clients on streets (such as cinema, park, bus-stand, railway station, hotel/lodge) and provided services at hotel/lodge or a place of client’s choice. The IBBA survey in Andhra Pradesh was led by the National Institute of Nutrition. The National AIDS Research Institute coordinated the conduct of the IBBA survey at the national level and FHI 360 provided technical assistance for implementing the IBBA. The ethics approval for this study was provided by the ethics committees of these institutions. No primary data were collected for this analysis, and we used data that did not have any personal identifiers.

### Data

#### *FSW behavioral data*

We used data on FSWs from the two rounds of IBBA survey for Andhra Pradesh, detailed methodology of which have been published elsewhere [12]. The primary objective of IBBA surveys was to collect data for assessing the outcomes and impact of interventions implemented as part of the *Avahan* program in Andhra Pradesh [12]. IBBA surveys were conducted in eight districts of Andhra Pradesh - Chittoor, East Godavari, Guntur, Hyderabad, Karimnagar, Prakasam, Vishakhapatnam and Warangal. The first round of IBBA survey was conducted during November 2005 - June 2006 and the second round during March - September 2009. A probability sampling method was used in all districts with conventional cluster sampling used to sample the brothel- and home-based FSWs, and time cluster sampling used to sample street-based FSWs. The sample sizes was 400 FSWs (all types) per district to track changes in key risk behaviours over time and for assessment of district level impact, in which FSWs aged 18 years or more who sold sex at least once in exchange for cash in the previous month were eligible to be interviewed. Data were collected through face-to-face interviews in a private location specifically set up for the interview and clinical examination. A total of 3,271 FSWs participated in IBBA survey round 1 and 3,225 in round 2 of whom 1,728 (52.8%) and 2,346 (72.7%) were street-based FSWs, respectively. This difference in proportions is thought to be due to a variable implementation of the sampling approach in the two rounds. The likely reason for this is that mapping of potential FSW sites was done based on information available from the local partners, and mapping information was updated by visiting each site. Additional seeds for respondent-driven sampling were selected during the conduct of the survey if the earlier seeds did not succeed in developing active recruitment chains.

#### *FSW HIV program data*

The computerised management information system (CMIS) of *Avahan* captures HIV program-related information for the *Avahan* districts [21,22]. We used data on the estimated numbers of FSWs in each of the eight districts; FSWs registered with targeted interventions (TIs), the estimated annual condom requirement, and annual free distribution of condom for FSWs for the years 2007 and 2008 from CMIS. We computed the mean count for these indicators from these two years of data.

For this analysis, we computed the proportion of FSWs registered with TIs from the total estimated number of FSWs in each IBBA district in Andhra Pradesh, and these were 58.1, 61.3, 83.5, 35.5, 101.9, 88.0, 75.4 and 88.9 for Chittoor, East Godavari, Guntur, Hyderabad, Karimnagar, Prakasam, Visakhapatnam and Warangal, respectively. Based on the median of these proportions, the IBBA districts were divided into two groups as “low” (≤75.4) and “high” groups for analysis. Similarly, we computed the proportion of condom requirement target met in each district as a proportion of the annual free distribution of condom for FSWs from the annual condom requirement which was 102.7, 133.2, 131.7, 36.8, 86.5, 128.1, 197.0 and 60.4 for Chittoor, East Godavari, Guntur, Hyderabad, Karimnagar, Prakasam, Visakhapatnam and Warangal respectively, and districts with ≥100 were grouped as “condom requirement target achieved” districts and the others as “condom requirement target not achieved” districts for analysis.

#### *District level contextual data*

We used data on total and urban population for each IBBA districts from the Census for years 2001 and 2011 [23,24]. We then projected total and urban population for each IBBA district exponentially for the IBBA survey years, 2006 and 2009 from the Census 2001 and 2011, respectively. From this projected total population for 2006 and 2009, we calculated the proportion of urban population for each IBBA district, and then computed the mean of urban population for these two years. Later, the districts were categorised into three equal groups based on the mean urbanisation proportion as low (≤19.4), medium (>19.4 and ≤28.8) and high (>28.8) proportion of urban districts. We had also considered district level adult literacy; however, the distribution of literacy in these districts was more or less similar to that of urbanisation. Therefore, only urbanisation was included in the analysis.

### Data analysis

SPSS 17.0 (IBM SPSS statistics standard, USA) and STATA 11.2 (StataCorp, USA) were used for data analysis which was restricted to only street-based FSWs from both the rounds of IBBA surveys. Appropriate weights were used for HIV prevalence estimation and bivariate and multivariate analysis was performed. Differentials in the sample of street-based FSWs between the two rounds of IBBA survey and HIV prevalence among street-based FSWs by districts are presented.

A multilevel logistic model was chosen to investigate factors associated with inter-district variations in positivity of HIV by fitting a two-level model, with individuals at level 1 nested within districts at level 2 by controlling for the IBBA round. Data from both IBBA rounds were used together in this model. Risk factors assessed at the individual level included age of FSW, marital status, condom use with regular sex partner and with occasional/regular clients, whether sex work was the main source of income for FSW, and history of violence/forced sex in last one year. For this analysis, the reported use of condom every time or most of the time was considered as consistent condom use with regular sex partner and with occasional/regular client. At the district level, proportion of FSWs contacted registered with TIs, condom requirement target met and proportion of urban population was used.

We constructed four models including random intercept model and random slope model. The first model, an empty model or unconditional model without any exposure variables was specified to decompose the amount of variance that existed at the district level. The second intercept model contained only individual-level variables and the third intercept model was extended to include the district level FSW programme related data (from CMIS) and level of urbanisation. Finally, we ran a random slope model allowing district level variation in the IBBA rounds. The results of fixed effect are presented as odds ratios (OR) with 95% confidence interval (CI), and of random effect as variance. We also examined the effect of IBBA rounds on HIV prevalence by the level of urban population in districts. We calculated the effect of urbanisation and IBBA survey rounds on HIV prevalence using the z test.

## Results

A total of 1,728 and 2,346 street-based FSWs participated in the IBBA round 1 and round 2 surveys, respectively (Table 1). The sample of street-based FSWs ranged from 36% in Guntur to 89% in Hyderabad with the median age of 29.9 years in round 1, from 50% in Visakhapatnam to 98.3% in Warangal in round 2 with the median age of 30.6 years. The difference in proportion of street-based FSWs in the total sample of FSWs was 20% or more between the two rounds in Chittoor, Guntur, East Godavari and Warangal districts. The overall HIV prevalence among street-based FSWs was 16.0% (95% CI 14.2 – 17.7%) and 12.9% (95% CI 11.5 – 14.2%) in round 1 and 2, respectively (p = 0.005, Table 1).

**Table 1:** Distribution of street-based female sex workers (FSW) and HIV prevalence among them by districts in the state of Andhra Pradesh from Integrated Behavioural and Biological Assessment (IBBA) Round 1 (R1) and Round 2 (R2) (weighted)

**District**	Total FSW sample*		**Street-based FSW N (%)**		**HIV prevalence among street-based FSW (%) [95% CI]**	
	**IBBA R1**	**IBBA R2**	**IBBA R1**	**IBBA R2**	**IBBA R1**	**IBBA R2**
Chittoor	401	398	147 (36.7)	252 (63.3)	8.9 [4.4 – 13.8]	11.9 [7.9-15.9]
East Godavari	422	401	208 (49.3)	287 (71.6)	29.8 [23.4 – 35.9]	28.2 [23.0 – 33.4]
Guntur	405	405	145 (35.8)	263 (64.9)	24.1 [16.7 – 30.8]	7.6 [4.4 – 10.8]
Hyderabad	399	401	354 (88.7)	392 (97.8)	14.4 [10.6 – 17.9]	9.9 [6.9 – 12.8]
Karimnagar	412	402	246 (59.7)	317 (78.9)	15.4 [10.8 – 19.9]	6.6 [3.9 – 9.5]
Prakasam	404	408	187 (46.3)	236 (57.8)	12.8 [7.9 – 17.4]	9.7 [5.9 – 13.5]
Vishakhapatnam	411	409	213 (51.8)	204 (49.9)	16.0 [11.1 – 21.0]	14.2 [9.6 – 19.2]
Warangal	417	401	228 (54.7)	394 (98.3)	8.8 [5.1 – 12.7]	15.0 [11.5 -18.6]
OVERALL	3,271	3,225	1,728 (52.8)	2,346 (72.7)	16.0 [14.2 – 17.7]	12.9 [11.5 – 14.2]

*Includes home, brothel and street-based FSW.

CI denotes confidence interval.

**Figure 1:**
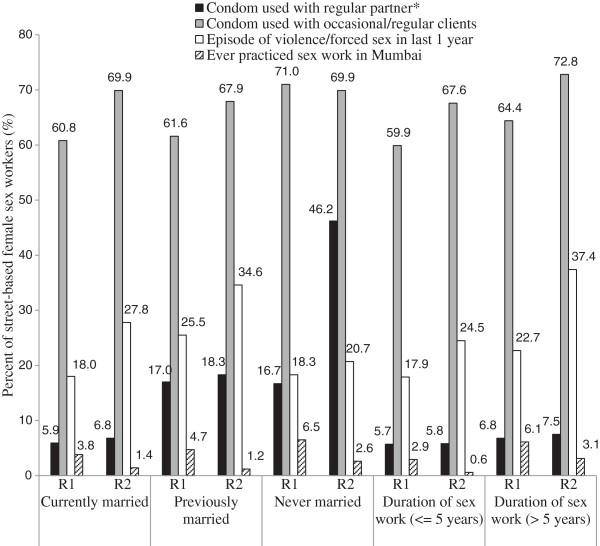
Distribution of select sex work-related risk behaviour variables for street-based female sex workers in the state of Andhra Pradesh from Integrated Behavioural and Biological Assessment Survey Round 1 (R1) and Round 2 (R2).

Significant differences in demography and sex-work related factors were found in the sample of street-based FSWs between the two rounds of IBBA survey. IBBA survey round 2 had a significantly higher proportion of never married FSWs (9%) and those reporting no regular sex partner (28.8%) as compared with 5.5% and 21.7% in round 1 (p < 0.001), respectively. The proportion of FSWs reporting practicing sex work ever in Mumbai had decreased over the two rounds (4.1% vs 1.4%; p < 0.001). The median duration of sex work decreased from 5 to 4 years whereas the number of clients per week increased from 6 to 9 clients from round 1 to round 2, respectively. A substantial increase was reported in consistent condom use with regular sex partner by never married FSWs from 16.7% in round 1 to 46.2% in round 2 (p < 0.001) and proportion of FSWs reporting an episode of violence/forced sex in last one year increased significantly with increasing duration of sex work in both rounds (p < 0.001) (Figure 1). Also, a significant increase in consistent condom use with regular/occasional clients was reported based on the duration of sex work in round 2 survey (p < 0.001).

The results of multilevel analysis for both the IBBA rounds combined are presented in Table 2. The district level variance was evaluated for the different characteristics of street-based FSWs. A comparison between the null model and the individual level random coefficient model indicated that the district level variation reduced to 17% from 21% in the null model. With the addition of FSW programme related indicators (from CMIS) and the proportion of urban population at district level in the model, the district level variation reduced to 2.7%, and the effects of individual characteristics remained unchanged after allowing for these district level indicators. FSWs aged 18–39 years (OR 1.93; 95% CI 1.39-2.68) and those previously married FSWs (OR 1.87; 95% CI 1.47-2.39), and an episode of violence/forced sex in the last one year (OR 1.74; 95% CI 1.42-2.14) were strongly associated with HIV positivity. IBBA round was a significant negative predictor for HIV positivity (OR 0.60; 95% CI 0.50-0.73). While allowing for the district level variables in the model, HIV positivity was significantly higher for districts with high proportion of FSWs registered with TIs (OR 2.02; 95% CI 1.18-3.45), and for districts with medium (OR 2.54; 95% CI 1.58-4.08) and high (OR 1.55; 95% CI 1.05-2.29) proportion of urban population. The odds of HIV positivity were significantly lower for districts which had met the condom requirement targets (OR 0.50; 95% CI 0.26-0.97). The random slope model showed the effect of IBBA round on HIV varied significantly and the estimated district level variance in HIV prevalence was 0.07 for IBBA round 2.

**Table 2:** Measures of variations and predictors for HIV prevalence among street-based female sex workers (FSW) by multilevel logistic regression for the two rounds combined of Integrated Behavioural and Biological Assessment survey (IBBA) (un-weighted data)

**Variable**	**Category**	**Total**	**HIV positive**	**Individual model**	**Individual + district level intercept model**	**Individual + district level slope model**
		**N = 3,873**	**N (%)**	**Odds of having HIV (95% CI)**	**Odds of having HIV (95% CI)**	**Odds of having HIV (95% CI)**
IBBA survey	Round 1	1688	285 (16.9)	1	1	1
	Round 2	2185	266 (12.2)	0.60 (0.50-0.73)	0.60 (0.49-0.72)	0.57 (0.43-0.75)
Age group	>40 years	470	49 (10.4)	1	1	1
	18-39 years	3403	502 (14.8)	1.93 (1.39-2.68)	1.95 (1.40-2.71)	1.95 (1.40-2.71)
Marital status	Currently married	2688	307 (11.4)	1	1	1
	Previously married	892	195 (21.9)	1.87 (1.47-2.39)	1.89 (1.48-2.42)	1.89 (1.48-2.42)
	Never married	289	49 (17.0)	1.23 (0.84-1.80)	1.26 (0.86-1.84)	1.25 (0.85-1.82)
Consistent condom use with regular sex partner	Yes	242	45 (18.6)	1	1	1
	No	2635	304 (11.5)	0.84 (0.58-1.21)	0.85 (0.59-1.22)	0.86 (0.60-1.25)
	No regular sex partner	996	202 (20.3)	1.21 (0.82-1.78)	1.22 (0.83-1.79)	1.24 (0.84-1.83)
Sex work as main source income	Yes	1777	288 (16.2)	1.22 (1.00-1.48)	1.22 (1.00-1.48)	1.22 (1.01-1.48)
	No	2096	263 (12.5)	1	1	1
Consistent condom use with occasional/regular clients	Yes	2557	390 (15.3)	1.29 (1.05-1.59)	1.27 (1.03-1.57)	1.25 (1.01-1.55)
	No	1316	161 (12.2)	1	1	1
Episode of violence/forced sex in last 1 year	Yes	960	189 (19.7)	1.74 (1.42-2.14)	1.76 (1.43-2.16)	1.74 (1.42-2.14)
	No	2904	361 (12.4)	1	1	1
Ratio of FSWs contacted and registered with targeted interventions	Low	2235	263 (11.8)		1	1
	High	1638	288 (17.6)		2.02 (1.18-3.45)	2.23 (1.28-3.89)
Condom requirement target met in district	No	1863	216 (11.6)		1	1
	Yes	2010	335 (16.7)		0.50 (0.26-0.99)	0.42 (0.21-0.84)
Proportion of urban population in district	Low	1555	149 (9.6)		1	1
	Medium	1178	235 (19.9)		2.54 (1.58-4.08)	2.17 (1.34-3.51)
	High	1140	167 (14.6)		1.55 (1.05-2.29)	1.28 (0.86-1.91)
District variance total				0.166 (0.093)	0.027 (0.024)	0.005 (0.087)
District variance IBBA round 2						0.070 (0.141)
LR test vs. logistic regression: chibar2(01)				53.80	3.75	7.23
Prob > =chibar2				<0.001	0.026	0.065

District level variables include FSW programrelated data and level of urbanisation. CI denotes confidence interval.

While examining the effect of IBBA survey rounds on HIV prevalence by the level of urban population in district, the survey round did not have a significant effect on HIV prevalence among street-based FSWs in districts with medium level of urban population (OR 0.76, p = 0.069). However, a significant negative effect was seen in the low and high urban proportion districts (OR 0.38, p < 0.001 and OR 0.68, p = 0.025, respectively). Also, districts with medium level urban population had significantly higher odds of HIV positivity among street-based FSWs in both IBBA survey rounds (OR 1.81, p = 0.023 for round 1 and OR 3.62, p < 0.001 for round 2).

**Figure 2:**
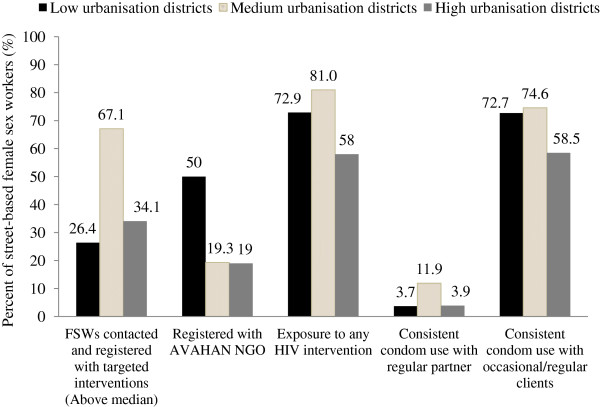
Distribution of select programme-related variables for street-based female sex workers in the state of Andhra Pradesh from Integrated Behavioural and Biological Assessment Survey Round 2 (R2).

The proportion of FSWs registered with TIs (Figure 2) was significantly higher in the medium level urbanisation districts (67.1%) as compared with the high and low urban districts (p < 0.001), and also the consistent condom use with regular partner was reported comparatively higher by FSWs residing in the medium level urbanization districts (p < 0.001). However, the proportion of FSWs registered with *Avahan* NGOs was the highest (50%) in the districts with low urbanisation as compared with the medium and high level urbanisation districts (p < 0.001).

## Discussion

In this paper we have described a multilevel approach to examine the contextual factors and aspects of the HIV intervention program which could be associated with HIV prevalence among street-based FSWs in the Indian state of Andhra Pradesh, while adjusting for individual level risk factors.

With this analysis of pooled IBBA data, we have documented findings that indicate the effect of HIV interventions in this population over this three-year period. Consistent condom use with regular/occasional clients increased between the two rounds, and low HIV positivity was documented in the districts where condom requirement for FSWs was met. It has been previously reported that the availability of condoms among FSWs increased through the distribution of free condoms under the *Avahan* program and had reached the estimated monthly requirement of condoms to cover the estimated number of commercial sex acts for each FSW [10]. Also, free distribution and social marketing of condom done under NACO facilitated the availability of more than sufficient condoms to FSWs [22,25,26]. This finding supports ensuring continuing availability of supply of condoms among street-based FSWs to further prevent HIV transmission.

HIV positivity was significantly higher in districts where a higher proportion of FSWs was registered with TIs. As TI strategy is based on the premise that prevention of HIV transmission from FSWs to their clients will likely result in lower rates of HIV transmission, it seems that many interventions reach FSWs who are already infected with HIV or are at high risk of HIV. This finding in the context of increased consistent condom use by FSWs over the three-year period suggest that the programmatic strategies of the TIs were in the expected direction to reduce HIV transmission from these FSWs to their clients. Accordingly, the higher HIV prevalence among FSWs in districts where a higher proportion of FSWs were registered with TIs does not reflect a negative impact of interventions; rather it likely reflects better targeting of FSWs at higher risk of HIV. Other analyses of the *Avahan* intervention have reported beneficial effect in reducing HIV in the general population, indicating that targeted interventions can help reduce overall transmission of HIV in India [15,16].

Higher HIV positivity was documented in districts with medium proportion of urban population, with no significant difference in HIV positivity over the three-year period in districts with medium urbanisation. This finding suggests a need to increase the focus of HIV interventions in such districts. We found evidence of increase in consistent condom use with clients among street-based FSWs between the two rounds of IBBA; however, similar increase was not seen for condom use with regular sex partners which is similar to what was reported previously [10,13]. Even though the *Avahan* program focused on increase in condom use with all types of sex partners of FSWs, the inconsistent condom use with regular sex partners indicates the need to increase the programme focus on this aspect of HIV prevention. At the individual level, FSWs aged 18–39 years, those previously married, those with sex work as main source income, and those who reported an episode of violence/forced sex were strongly positively associated with HIV positivity. Sex work due to economic needs resulting from various reasons among FSWs [3], and a high prevalence of violence and evidence of related increase in HIV risk among FSWs have been previously reported [26-30].

There are several limitations that need to be considering while interpreting the findings reported in this paper. As highlighted, there were some socio-demographic differences in the FSW samples between the two rounds of IBBA, which may have influenced to some degree the differences observed in the two rounds. In addition, self-reported data on condom use by FSWs may be influenced more by social desirability bias in second round of IBBA as they could have been aware of the expectation of increased condom use and hence could have over-reported it. A significant limitation of this analysis is that causality between the *Avahan* program and the decrease in HIV positivity and the increase in condom use by FSWs observed after its roll-out cannot be ascertained in a pre and post study design. In addition, other potential confounders related to interventions or context, which could not be included in the analysis, may have also influenced the changes in behaviour and HIV positivity. However, other recent analyses have indicated that the *Avahan* interventions for high risk groups have led to reduction of HIV in the general population in several states of India [15,16].

## Conclusions

An overall declining trend in HIV prevalence among street-based FSWs was reported after implementation of Avahan intervention in Andhra Pradesh state in southern India. Variations in HIV prevalence at the district level were observed. The data presented in this paper provide further evidence of a beneficial effect of *Avahan* program, and point to some important contextual factors along with individual factors that could be addressed to further reduce HIV transmission in street-based sex workers in India.

## Abbreviations

CI: Confidence interval; CMIS: Computerised management information system; FHI: Family health international; FSWs: Female sex workers; IBBA: Integrated biological and behavioural assessment; NACO: National aids control organization; OR: Odds ratio; TIs: Targeted interventions.

## Competing interests

The authors declare that they have no competing interests.

## Authors’ contributions

RD, GAK and LD led the design and interpretation. GAK and RD drafted the manuscript. GAK did the statistical analysis. MA contributed to the interpretation of findings. All authors approved the final version of the manuscript.

## Pre-publication history

The pre-publication history for this paper can be accessed here:

http://www.biomedcentral.com/1472-6874/14/65/prepub

## References

[B1] National AIDS Control Organization (NACO) Auual Report 2011–12 2012 New Delhi: NACO, Ministry of Health and Family Welfare Government of India; 2012. Available at. http://www.naco.gov.in/upload/Publication/Annual%20Report/NACO_AR_Eng%202011-12.pdf

[B2] Dandona R Dandona L Gutierrez JP Kumar GA McPherson S Samuels F Bertozzi SM ASCI FPP Study Team: High risk of HIV in non-brothel based female sex workers in India BMC Public Health 2005 5 87 10.1186/1471-2458-5-87 16111497 PMC1208909

[B3] Dandona R Dandona L Kumar GA Gutierrez JP McPherson S Samuels F Bertozzi SM ASCI FPP Study Team: Demography and sex work characteristics of female sex workers in India BMC Int Hum Rights 2006 6 5 10.1186/1472-698X-6-5 PMC1468426 16615869

[B4] Shahmanesh M Wayal S Targeting commercial sex-workers in Goa, India: time for a strategic rethink? Lancet 2004 364 9442 1297 1299 10.1016/S0140-6736(04)17206-1 15474122

[B5] Shannon K Kerr T Allinott S Chettiar J Shoveller J Tyndall M Social and structural violence and power relations in mitigating HIV risk of drug-using women in survival sex work Soc Sci Med 2008 66 4 911 921 10.1016/j.socscimed.2007.11.008 18155336

[B6] Bill & Melinda Gates Foundation Avahan—The India AIDS Initiative The business of HIV prevention at scale 2008 New Delhi: Bill & Melinda Gates Foundation Available at. http://www.gatesfoundation.org/avahan/Documents/Avahan_HIVPrevention.pdf

[B7] National AIDS Control Organization (NACO) Auual Report 2010–11 2011 New Delh: NACO, Ministry of Health and Family Welfare Government of India; 2011. Available at. http://www.naco.gov.in/upload/REPORTS/NACO%20Annual%20Report%202010-11.pdf

[B8] Gurnani V Beattie TS Bhattacharjee P Team CFAR Mohan HL Maddur S Washington R Isac S Ramesh BM Moses S Blanchard JF An integrated structural intervention to reduce vulnerability to HIV and sexually transmitted infections among female sex workers in Karnataka state, south India BMC Public Health 2011 2 11 755 21962115 10.1186/1471-2458-11-755 PMC3205062

[B9] Jana S Basu I Rotheram-Borus MJ Newman PA The sonagachi project: a sustainable community intervention AIDS Educ Prev 2004 16 5 405 414 10.1521/aeap.16.5.405.48734 15491952

[B10] Rachakulla HK Kodavalla V Rajkumar H Prasad SPV Kallam S Goswami P Dale J Adhikary R Paranjape R Brahmam GNV Condom use and prevalence of syphilis and HIV among female sex workers in Andhra Pradesh, India – following a large-scale HIV prevention intervention BMC Public Health 2011 11 Suppl 6 S1 10.1186/1471-2458-11-S6-S1 22376071 PMC3287547

[B11] Ramakrishnan L Gautam A Goswami P Kallam S Adhikary R Mainkar MM Ramesh BM Morineau G George B Paranjape R Programme coverage, condom use and STI treatment among FSWs in a large-scale HIV prevention programme: results from cross–sectional surveys in 22 districts in southern India Sex Transm Infect 2010 86 Suppl 1 i62 i68 10.1136/sti.2009.038760 20167734 PMC3252617

[B12] Mainkar MM Pardeshi DB Dale J Deshpande S Khazi S Gautam A Goswami P Adhikary R Ramanathan S George B Paranjape R Targeted interventions of the Avahan program and their association with intermediate outcomes among female sex workers in Maharashtra, India BMC Public Health 2011 11 Suppl 6 S2 10.1186/1471-2458-11-S6-S2 22375562 PMC3287555

[B13] Thilakavathi S Boopathi K Kumar CPG Santhakumar A Senthilkumar R Eswaramurthy C Bharathy LV Ramakrishnan L Thongamba G Adhikary R Paranjape R Assessment of the scale, coverage and outcomes of the Avahan HIV prevention program for female sex workers in Tamil Nadu, India: is there evidence of an effect? BMC Public Health 2011 11 Suppl 6 S3 10.1186/1471-2458-11-S6-S3 22375609 PMC3287556

[B14] Indian Council for Medical Research (ICMR) Family Health International (FHI) Integrated Behavioural and Biological Assessment — National Summary Report, India (Round 2):2009–2010 National Summary Report 2011 New Delhi: ICMR and FHI Available at. http://www.fhi360.org/sites/default/files/media/documents/

[B15] Ng M Gakidou E Levin-Rector A Khera A Murray CJ Dandona L Assessment of population-level effect of Avahan, an HIV- prevention initiative in India Lancet 2011 378 9803 1643 1652 10.1016/S0140-6736(11)61390-1 21993161

[B16] Pickles M Boily M Vickerman P Lowndes CM Moses S Blanchard JF Deering KN Bradley J Ramesh BM Washington R Adhikary R Mainkar M Paranjape R Alary M Assessment of the population-level effectiveness of the Avahan HIV-prevention programme in South India: a preplanned, causal-pathway-based modelling analysis Lancet Glob Health 2013 1 5 e289 e299 10.1016/S2214-109X(13)70083-4 25104493

[B17] Boerma JR Weir SS Integrating demographic and epidemiological approaches to research on HIV/AIDS: The proximate-determinants frame work J Infect Dis 2005 191 Suppl 1 S61 S67 15627232 10.1086/425282

[B18] Nzyuko S Lurie P McFarland W Leyden W Nyamwaya D Mandel JS Adolescent sexual behavior along the Trans-Africa highway in Kenya AIDS 1997 11 Suppl 1 S21 S26 9376097

[B19] Oyefara JL Food insecurity, HIV/AIDS pandemic and sexual behaviour of female commercial sex workers in Lagosmetropolis, Nigeria SAHARA J 2007 4 2 626 635 10.1080/17290376.2007.9724884 18071614

[B20] Ramesh BM Moses S Washington R Isac S Mohapatra B Mahagaokar SB Adhikary R Brahmam GN Paranjape R Subramanian T Blanchard JF IBBA study team: determinants of HIV prevalence among female sex workers in four south Indian states: analysis of cross-sectional surveys in twenty-three districts AIDS 2008 22 Suppl 5 S35 S44 10.1097/01.aids.0000343762.54831.5c 19098478

[B21] Bill & Melinda Gates Foundation Use it or Lose it How Avahan Used Data To Shape Its Hiv Prevention Efforts In India 2008 New Delhi: Avahan Available at. http://www.gatesfoundation.org/avahan/Documents/Avahan_UseItOrLooseIt.pdf

[B22] National AIDS Control Organization (NACO) Annual CMIS bulletin 2008–2009 2010 New Delhi: NACO, Ministry of Health and Family Welfare Government of India; 2010. Available at. http://naco.gov.in/upload/HIV%20data/NACO%20CMIS%20BULLETIN%202008-09.pdf

[B23] Registrar General of India Census of India 2001 Primary Census Abstract Andhra Pradesh 2001 New Delhi: Office of the Registrar General of India Available at. http://www.censusindia.gov.in/Tables_Published/Basic_Data_Sheet.aspx

[B24] Registrar General of India Census of India 2011 Primary Census Abstract Andhra Pradesh 2011 New Delhi: Office of the Registrar General of India Available at. http://censusindia.gov.in/2011-prov-results/prov_data_products_andhra.html

[B25] National AIDS Control Organization (NACO) Condom promotion. National AIDS control programme, phase-III, India 2011 New Delhi: NACO, Ministry of Health and Family Welfare, Government of India Available at. http://www.naco.gov.in/upload/IEC%20Division/Parliamentarian%20Forum%204-5%20july%202011/Condom%20Monograph.pdf

[B26] Piot B Mukherjee A Navin D Krishnan N Bhardwaj A Sharma V Marjara P Lot quality assurance sampling for monitoring coverage and quality of a targeted condom social marketing programme in traditional and non-traditional outlets in India Sex Transm Infect 2010 86 Suppl 1 i56 i61 10.1136/sti.2009.038356 20167732 PMC3252603

[B27] George A Sabarwal S Martin P Violence in contract work among female sex workers in Andhra Pradesh, India J Infect Dis 2011 204 Suppl 5 S1235 S1240 10.1093/infdis/jir542 22043038

[B28] Karnataka Health Promotion Trust (KHPT) Population Council: Patterns of migration/mobility and HIV risk among female sex workers 2008 Karnataka. Bangalore: KHPT Available at. http://www.popcouncil.org/pdfs/India_FSWMigrantHIVKarnataka.pdf

[B29] Panchanandeswaran S Johnson SC Sivaram S Srikrishnan AK Latkin C Bentley ME Solomon S Go VF Celentano D Intimate partner violence is as important as client violence in increasing street-based female sex workers’ vulnerability to HIV in India Int J Drug Policy 2008 19 2 106 112 10.1016/j.drugpo.2007.11.013 18187314 PMC2423812

[B30] Reed E Gupta J Biradavolu M Blankenship KM Migration/mobility and risk factors for HIV among female sex workers in Andhra Pradesh, India: implications for HIV prevention Int J STD AIDS 2012 23 4 e7 e13 10.1258/ijsa.2009.009421 22581964

